# Deoxycholic acid treatment for HIV-associated dorsocervical fat pad

**DOI:** 10.1016/j.jdcr.2026.03.030

**Published:** 2026-03-20

**Authors:** Jake Nusynowitz, Andrea Cespedes Zablah, Seth L. Matarasso

**Affiliations:** a Department of Dermatology, Florida International University Herbert Wertheim College of Medicine, Miami, Florida; b Department of Dermatology, Massachusetts General Hospital, Boston, Massachusetts; c Department of Dermatology, University of Illinois, Chicago School of Medicine, Chicago, Illinois; d Department of Dermatology, UCSF School of Medicine, San Francisco, California

**Keywords:** buffalo hump, deoxycholic acid, dorsocervical fat pad, HIV lipodystrophy, intralesional injection

## Introduction

HIV-associated lipodystrophy is a complication of early antiretroviral therapy, encompassing both lipoatrophy and lipohypertrophy. Dorsocervical fat pad enlargement, colloquially known as a “buffalo hump,” is a manifestation of lipohypertrophy that can cause pain, functional impairment, and social stigma. Although lipohypertrophy is reported in up to 40% of patients on early regimens, involvement of the dorsocervical fat pad occurs in only 2% to 19% of cases. Zidovudine and protease inhibitor exposure are strongly implicated in its development.^1^

Surgical approaches, such as liposuction or direct lipectomy, have been the standard treatment; however, recurrence and variable outcomes are common.^2^^,^^3^ To date, no medical therapy has been established. We report on the use of intralesional deoxycholic acid (Kybella, Allergan, AbbVie Corporation) for the reduction of HIV-associated dorsocervical fat pad, a technique not previously described in the dermatology literature.

## Case description

A 71-year-old White male diagnosed with HIV more than 30 years ago presented for evaluation of a persistent dorsocervical fat pad. His HIV status had been well controlled on emtricitabine/rilpivirine/tenofovir alafenamide, with stable CD4 counts ranging between 500 and 600/μL. His fat pad first developed at age 50 while receiving zidovudine-based therapy.

He underwent 2 tumescent liposuction procedures performed 3 years apart, which only resulted in partial resolution. The patient subsequently received intra-abdominal injections; although he did not recall the specific medication, he reported that the agent was intended to reduce fat deposition. Based on this description, Egrifta SV/Egrifta WR (Tesamorelin, Theratechnologies Inc), a growth hormone–releasing analog, was the most likely agent used.^4^ Eight years later, he underwent direct surgical excision under general anesthesia, which provided moderate improvement. At that time, histopathologic examination demonstrated adipocyte hypertrophy with associated vascularization and fibrosis, findings consistent with dorsocervical fat pad enlargement. Despite these interventions, a large, painful subcutaneous residual mass persisted.

**Fig 1:**
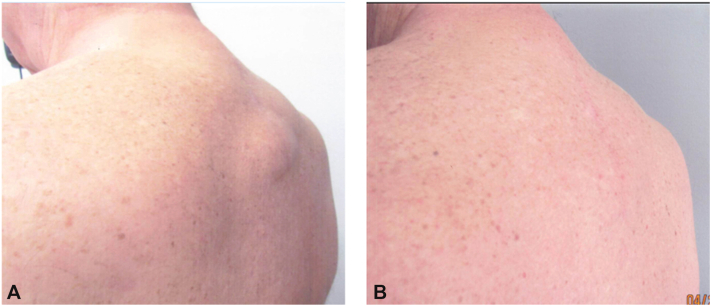
The patient’s midline dorsocervical fat deposition attributed to antiretroviral therapy, extending from approximately the third cervical to the third thoracic vertebrae. **A,** Before and **(B)** after 3 treatment sessions performed at 3-month intervals using 2 cc per treatment (total volume, 6 cc) of deoxycholic acid.

Overlying a well-healed 20.0 cm linear scar, physical examination revealed a 15 × 14 × 3 cm (length × width × height) soft, freely mobile subcutaneous mass located in the midline thoracic spine. Aside from the surgical cicatrix, no additional cutaneous abnormalities were noted (Fig 1, *A*). Given that both the clinical presentation and prior histopathology were consistent with a dorsocervical fat pad secondary to antiretroviral therapy, the patient declined further diagnostic workup or imaging. Although he expressed cosmetic concerns, his primary complaints were difficulty lying flat and physical discomfort related to the size of the mass.

The patient’s comorbidities included hypertension and peripheral neuropathy. He had no history of oral or systemic glucocorticoids, and there was no history of Cushing’s syndrome or long-term exogenous steroid exposure that would predispose him to the formation of the fat pad. Given the lack of durable benefit from prior surgeries and the patient’s desire to avoid further operative interventions, intralesional deoxycholic acid was offered as an off-label therapeutic trial.

Without altering the product provided by the manufacturer, 3 treatments were performed. Two cc of deoxycholic acid (10 mg/mL) were injected directly into the subcutaneous fat pad using a one-inch 30-gauge needle at each session. Sessions were spaced 3 months apart. The patient achieved a significant reduction in hump size after the first session and complete resolution after the third (Fig 1, *B*). He reported improved comfort, posture, and fit of clothing. Adverse events were limited to mild burning upon injection, with no functional complications reported. Thirty-six months after the final injection, the patient did not report recurrence. After marked clinical reduction of the mass, a punch biopsy of the treated area was performed to evaluate histologic changes. Histopathologic examination demonstrated a reduction in adipocyte volume and number, accompanied by fibrosis and septal thickening.

## Discussion

Dorsocervical fat pads are 1 manifestation of HIV-associated lipohypertrophy with both functional and psychosocial consequences.^1^^,^^5^ Patients may experience pain, impaired neck mobility, clothing fit issues, and stigmatization due to visible changes in body habitus. Surgical options remain invasive and prone to recurrence.^2^^,^^3^ Alternative injection-based approaches have been attempted. One case reported a significant improvement using an unknown adipocytolytic aqueous solution with a sustained response at 18 months.^6^

Deoxycholic acid is Food and Drug Administration–approved solely for submental fat reduction. It functions by lysing adipocytes and promoting fibrotic remodeling that helps tighten skin. As a naturally occurring bile acid involved in dietary fat solubilization, it has been shown in culture to selectively induce adipocytolysis without harming skin or muscle. Clinical trials of the synthetic formulation have confirmed its safety and efficacy for localized fat reduction, and its off-label use in other small fat deposits such as axillary breast tissue or thighs has been documented.^7^ In the single study evaluating deoxycholate use in HIV-positive patients with lipohypertrophy, treatment regimens were not specified, most patients required 6 to 8 sessions, treated areas were not isolated, and long-term follow-up was not reported.^8^

This case highlights a novel off-label application of deoxycholic acid injection for dermatologists treating HIV-associated dorsocervical fat pads. As an ambulatory procedure, it represents a well-tolerated option either as initial therapy or for patients with persistent adiposity who have not responded to standard treatments or who are not appropriate surgical candidates. Suggested treatment parameters and long-term follow-up in this case demonstrate sustained improvement. This technique is readily performed in the outpatient dermatology setting and has the potential to significantly improve quality of life for patients affected by this disfiguring and painful condition.

## Conflicts of interest

None disclosed.
